# Symptoms Associated with Low Threshold Lead Poisoning Among Roadside and Organized Panel Beaters in Enugu Metropolis, Nigeria

**DOI:** 10.5696/2156-9614-11.29.210303

**Published:** 2021-02-25

**Authors:** Chukwukasi Wilson Kassy, Chukwueloka Kingsley Uchegbu, Tuman Juliette Ango

**Affiliations:** Department of Community Medicine, University of Nigeria Teaching Hospital, Ituku/Ozalla, Enugu, Nigeria

**Keywords:** lead poisoning, low threshold, low dose exposures, symptoms, Enugu, Nigeria

## Abstract

**Background.:**

There is no safe level of human exposure to lead (Pb). Detecting common early symptoms of low threshold Pb poisoning can help to prevent the damaging effects of higher doses and chronic low dose exposures. Panel beaters or auto body mechanics are exposed to Pb through their occupational duties.

**Objectives.:**

The present study aimed to determine common early symptoms associated with lower threshold Pb poisoning among roadside and organized panel beaters in Enugu Metropolis, Nigeria.

**Methods.:**

This was a comparative cross-sectional study of 428 panel beaters in Enugu metropolis. A multi-stage sampling method was used to select 214 respondents each from the roadside and organized sectors. A semi-structured interviewer-administered questionnaire was used for data collection. Samples were collected under aseptic procedures. Blood Pb samples were extracted using the conventional wet acid digestion method and analyzed using a flame atomic absorption spectrometer (wavelength 283.3 nm). Comparative analysis was performed using the chi – square and Mann-Whitney U test. Statistical significance was set at P < 0.05.

**Results.:**

Median Pb levels were 3.0 ug/dL and 16.0 ug/dL among roadside and organized panel beaters, respectively, with a significant difference. Numbness of limbs (P = 0.010) and fatigue (χ^2^ = 5.294, P = 0.023) were found to be associated with roadside panel beaters, while weakness (χ^2^ = 6.185, P = 0.019) and fatigue (χ^2^ = 4.206, P = 0.046) were associated with organized panel beaters.

**Conclusions.:**

Nonspecific constitutional symptoms were common early symptoms of Pb poisoning irrespective of workplace occupational practices. These symptoms will help in early detection and control of occupational lead exposures.

**Participant Consent.:**

Obtained

**Ethics Approval.:**

Ethics approval was obtained from the Health Research Ethics Committee of the University of Nigeria Teaching Hospital, Ituku Ozalla, Enugu.

**Competing Interests.:**

The authors declare no competing financial interests.

## Introduction

Panel beaters and auto body mechanics are automobile technician subspecialties whose occupational practices include repair, cutting, soldering, welding and spray painting which exposes them to lead (Pb) poisoning. Other practices include repair of chips and scuffs, polishing and waxing, glass and interior repair, and wheel refinishing.^1^ Panel beaters operate in both the informal sector (operating along roadsides) and the formal organized sector. Organized panel beaters are those working in sectors with rules and requirements set by the government and the sector is governed by acts such as the Factories Act,^2^ labor laws, and Employee Compensation Act;^3^ they practice with safety precautions and have fixed working hours. The informal sector is a private unincorporated enterprise operating along roadsides with no regular system of data availability.^4^

Toxicities manifest as overt clinical symptoms primarily detected at high doses, thereby underestimating the actual burden of Pb toxicity.^5^ They occur in severe multi–systemic clinical forms: abdominal colic, joint and muscle pain, wrist and ankle drop, fine tremors, diminished visual intelligence and motor coordination, short term memory loss, irritability, encephalopathy, lethargy, delirium, convulsions, coma, fatigue, weakness, excessive tiredness, constipation, anorexia, persistent vomiting, impotence, infertility and reduced sex drive.^1,6,7,8,9^ These toxicities are common at blood lead levels of 40 μg/dL and above.^10,11^ The American Conference of Governmental Industrial Hygienists (ACGIH) has stated that blood lead levels (BLLs) should be controlled at 20 ug/dL.^12^

Low Pb levels refer to exposure levels below which Pb poisoning is apparent, non-specific, asymptomatic or subclinical, but not without harmful effects.^13^ Prevention measures and campaigns that lead to the removal of leaded paints in 1921 and leaded gasoline in the 1980s–90s resulted in significant lowering of Pb levels.^1,14^ In 1978, the United States Occupational Safety and Health Administration (OSHA) required interventions for adult workers at a BLL of 10 μg/dL and above.^15^ In 2012, the Centers for Disease Control and Prevention (CDC) set a threshold for elevated BLL for children at 5 μg/dL.^16^ Despite progress in the control of occupational Pb exposures and reduction of Pb exposures in work environments, studies have found the health effects of chronic exposures to Pb at lower doses or thresholds to include hypertension, a decrease in renal function, cognitive dysfunction and other nonspecific symptoms.^17,18^ This indicates that there is no safe level of Pb exposure, but as Pb levels increase, the severity of damage to target organs and consequent symptoms also increases.^6^

The World Health Organization (WHO) identifies Pb as one of 10 chemicals of public health concern.^13^ An estimated 120 million people around the world have BLLs greater than 10 ug/dL.^19^ In 2017 the Institute of Health Metrics and Evaluation (IHME) attributed 1.06 million deaths and 24.4 million disability-adjusted life years (DALYs) worldwide to Pb exposure.^13,20,21^ In Nigeria, Pb toxicity accounts for 1.6 deaths per 100 000 and 46.82 DALYS per 100 000 population.^20,21^ The IHME also estimated that in 2016, Pb exposure accounted for 63.2% of the global burden of idiopathic developmental intellectual disability, 10.3% of the global burden of hypertensive heart disease, 5.6% of the global burden of ischemic heart disease and 6.2% of the global burden of stroke, with the highest burden in developing countries.^13,20,21^ Even with severe underreporting, a study in the USA found that reducing occupational Pb limits produces annual societal benefits of almost $40,000 per highly exposed worker in the USA, taking into account the effects of direct and indirect costs and omitting health effects.^22^

**Abbreviations**
*BLL*: Blood lead levels

There is increasing scientific evidence of the harmful effects of Pb poisoning in nonspecific subclinical forms in addition to specific clinical forms. The present study will add to the existing literature on early common symptoms of Pb poisoning at reduced thresholds, thereby preventing the damaging health effects of low dose chronic exposure and high dose toxicities in low-income countries or industries where routine Pb monitoring may be difficult. This study will further emphasize the benefits of regular screening, and early diagnosis and treatment to mitigate this occupational hazard. For the most part, the diagnosis of Pb poisoning in adult workers is based on integration of data obtained from patient history, physical examination, laboratory tests and tests of specific organ function. This will help to prevent the development of higher BLLs above which overt severe clinical symptoms are seen.

The aim of the present study is to examine common symptoms associated with Pb poisoning at lower thresholds among roadside and formal sector panel beaters in Enugu Metropolis, Nigeria.

## Methods

The study area was Enugu metropolis which is the capital of Enugu State in the southeast geo–political zone of Nigeria. The metropolis is constituted by three Local Government Areas in Enugu State which are Enugu North, Enugu South and Enugu East and is inhabited primarily by the Igbo ethnic group.^23^ According to the 2006 National Population census, Enugu Metropolis had a total population of 722,664, representing 22.2% of the Enugu State population and 0.51% of the total Nigerian population.^23,24^ The main occupation is trade, followed by services like transportation and public service.^25^ The transportation business includes workshops where panel beaters engage in vehicle body repair. Roadside panel beaters operate mainly alongside roads and close to vehicle spare parts markets. They operate as unions, without government regulations and with no standard safety practices, while the organized panel beaters are governed by government acts like factory and labor acts, worker compensation laws, and standard safety precautions.

This was a comparative cross-sectional study of roadside and organized panel beaters in Enugu metropolis conducted from November 2018 to April 2019. Panel beaters and trainees who had spent over one year in the occupation and who were willing to participate in the study were included, while those on chelation therapy, on allopurinol, were severely ill or with chronic diseases were excluded.

The minimum sample size for the study was determined using the formula for comparing two independent proportions^26^ and using a prevalence of 14.3% for Nigerian adults with BLLs > 20 μg/dL from a previous study.^27^ A minimum sample size of 214 per sector was obtained after correcting for non-response rate, giving a total of 428 panel beaters.

A multistage sampling technique was used for both roadside and organized panel beaters in the present study. For the roadside panel beaters, the first stage was selection of Enugu North among the three local government areas (LGA) by simple random sampling using the balloting method. The second stage was selection of one division out of the five divisions in Enugu North LGA by simple random sampling using the balloting method. The third stage was selection of 10 branches out of the 13 branches in Enugu North LGA by simple random sampling using the balloting method. Lastly, stratification and proportionate allocation of panel beaters from all the workshops within the selected branches was performed based on the density of workers and workshop concentration. There were 29 small (<5 panel beaters), 16 medium (5–10 panel beaters) and 9 large (>10 panel beaters) workshops respectively from the 54 workshops. Using proportionate allocation, a total of 228 panel beaters were selected [2X29] + [5X16] + [10X9].

For organized panel beaters, the first stage was same as that for roadside workers. The second stage was proportionate allocation of government and private owned company workers using a ratio of 1:3. Fifty-six (56) panel beaters from government-owned workshops were selected out of 70 panel beaters by simple random sampling using the balloting method. The privately owned workshops were categorized into 10 small (2–4 panel beaters), 16 medium (5–7 panel beaters) and 9 large (8–12 panel beaters) workshops. One hundred and seventy (170) panel beaters were selected [2X10] + [5X16] + [10X9]. A total of 226 panel beaters were selected from the organized panel beaters taking into consideration missed and incomplete responses. Panel beating is a male-dominated subspecialty of automobile repairs, hence all our respondents were male.

An interviewer-administered semi-structured questionnaire was used to collect data from the roadside and organized panel beaters. The questionnaire was adapted from the medical evaluation questionnaire for occupational Pb exposure by the Massachusetts division of occupational safety *(Supplemental Material).*^28^

The questionnaire was pre-tested in Enugu East. About 20 samples were collected from both sectors for the pretest. The observed shortcomings in relevance and scope of questions were corrected before final administration of the questionnaires to the respondents. Data and samples were collected using research assistants comprised of three resident doctors and three phlebotomists. They were trained for two days, for two hours per day on sample collection procedures and questionnaire administration, communication and follow-up skills, study objectives and ethical issues involved in the research. Blood for Pb sampling was collected under aseptic procedures and analyzed at the biochemical laboratory unit of the Project Development Institute (PRODA), Enugu using a flame atomic absorption spectrometer at 283.3 nm wavelength. The PRODA is an industrial research institute under the Nigerian Federal Ministry of Science and Technology operating under a quality management system. Blood sample collection was done in an enclosed well-screened location. The venipuncture system was used to perform venipuncture and 2–3 ml of blood drawn into an edetic acid (EDTA) vacutainer bottle for BLL estimation. The samples were transported immediately to the laboratories using a Giostyle cold box (Giostyle, Urgnano, Italy) after each day, accompanied by 5- and 10-ml syringes, bleach, and gloves for maintenance of universal precautions. The blood samples were diluted to 10 ml using deionized water to obtain reliable results. The diluted samples were acidified with trichloroacetic acid (TCA) and Pb was extracted using a conventional wet acid digestion method. The aspirated extracts were analyzed by a flame atomic absorption spectrometer at a wavelength of 283.3 nm. Process controls involving quality of testing, sample handling, standard safety practices, verification and validation of results were ensured by the laboratory for quality control. The analytical accuracy was checked using an internal quality assessment method through machine calibration with a standard Pb stock solution, standardization of machine and appropriate wavelength setting. Data were entered and analyzed using the Statistical Package for the Social Sciences (SPSS) version 20.

Categorical variables were summarized using frequencies and proportion, while continuous variables were summarized using median and range for skewed data. Blood lead levels were categorized using a cut-off of < 10 μg/dl (unexposed), 10–40 μg/dl (normal, acceptable) and >40 μg/dl (dangerous), respectively.^15,29^ The proportion of panel beaters with a BLL of 10 ug/dL and above was determined. Comparison of variables was managed using the Mann Whitney U–test and Chi–square test. Level of significance was set at 0.05.

### Ethics approval

Ethics approval was obtained from the Health Research Ethics Committee of the University of Nigeria Teaching Hospital, Ituku Ozalla, Enugu. Permission was obtained from unions of panel beaters and organized panel beaters in Enugu State. Informed consent was obtained from participants. The outcomes of the analyses were relayed to the workers immediately. Some were informed by telephone while some results were delivered to their workplace by hand. Those with high blood levels were counseled and educated on the implications of their results, exposure sources, and safety and preventive measures. Those with BLLs between 10–40 μg/dL were told to retest in 6 months, those with BLLs 40–50 μg/dL were told to retest in 2 months, while those with BLLs > 50 μg/dL were advised to stop their occupational exposure and retest in one month. Chelation is recommended at BLL > 45 μg/dL.

## Results

The mean ages (±standard deviation) were 31.1 ± 10.3 years and 37.9 ± 12.1 years for roadside and organized panel beaters, respectively. All the respondents from both sectors were male and from the Igbo ethnic group. The majority of the respondents from the roadside and organized sectors had a secondary education. Many of the roadside panel beaters were single compared to organized panel beaters who were more likely to be married. Roadside workers tended to earn more than their counterparts in the organized sector *(Table 1).*

**Table 1:** Sociodemographic Characteristics of Roadside and Organized Panel Beaters

***Variable***	***Roadside panel beaters N = 214***		***Organized panel beaters N = 214***		***Statistical analysis***	
	Frequency	Percent	Frequency	Percent	**Chi square**	**P value**

***Age***						
*<30*	121	62.4	73	37.6		
*31–50*	81	44.0	103	56.0	28.027	<0.001*
*>50*	12	24.0	38	7.0		
					**T test**	**P value**
*Mean*	31.1		37.9		−6.26	<0.001*
*Standard deviation*	10.3		12.1			
					**Chi square**	**P value**
***Educational level***						
*Primary or none*	55	45.5	66	54.5		
*Secondary*	151	55.5	121	44.5		0.001*
*Tertiary*	8	22.9	27	77.1		
***Marital status***						
*Single*	128	61.8	79	38.2	22.463	<0.001*
*Married*	86	38.9	135	61.1		
***Monthly income*** *(#,Nigerian currency; $ USD)*						
*<#35000 (<$100)*	58	35.2	107	64.8	23.681	<0.001*
*>#35000 (> $100)*	156	59.3	107	40.7		

^*^ Significant

The most common occupational practice was panel beating or auto repair, while spray painting was the least common. The majority of the workers worked more than 8 hours per day, with roadside workers working more hours compared to the organized sector. Most workers in the roadside sector worked 2–3 days per week, while the organized sector worked more than 3 times per week. The majority of workers in both sectors had worked for 1–10 years *(Table 2).*

**Table 2:** Workplace Characteristics of Roadside and Organized Panel Beaters

***Variable***	***Roadside panel beaters***		***Organized panel beaters***		***Statistical analysis***	
	Frequency	Percent	Frequency	Percent	**Chi-square**	**P value**

***Occupational practice***						
*Panel beating*	204	95.3%	211	98.6%	3.887	0.049*
*Cutting metal*	163	76.2%	206	96.3%		
*Welding*	100	46.7%	140	65.4%		
*Soldering*	63	29.4%	100	46.7%		
*Spraying / painting*	59	27.6%	88	41.1%		
***Working hours / day***						
*<8 hours/day*	15	7.0%	47	22.0%	19.314	<0.001*
*>8 hours/day*	199	93.0%	167	78.0%		
***Frequency of work***						
*2–3 times/wk*	168	78.5%	0	0.0%	276.5	<0.0001*
*>3 times/wk*	46	21.5%	214	100.0%		
***Duration of work experience***						
*1 – 10 years*	125	58.4%	105	49.1%	5.995	0.050
*11 – 20 years*	56	26.2%	57	26.6%		
*21 – 50 years*	33	15.4%	52	24.3%		

^*^ Significant

The median BLL for the roadside and organized panel beaters was 3.0 μg/dL and 16.0 μg/dL, respectively. The prevalence of BPb at 10 μg/dl and above was 36.9% and 64.5% for roadside and organized panel beaters, respectively. The differences between the two groups were found to be significant. The majority of the panel beater BLLs were within the acceptable limits for occupational workers, although they were greater among the organized group *(Table 3).* Among the roadside panel beaters, numbness and fatigue were symptoms associated with BLLs of ≥ 10 μg/dL, while weakness and fatigue were associated with BLLs of ≥ 10 μg/dL among organized panel beaters *(Table 4).*

**Table 3:** Prevalence of Blood Lead Levels over 10 μg/dL Among Roadside and Organized Panel Beaters in Enugu Metropolis

***Variables***	***Roadside panel beaters N = 214***		***Organized panel beaters N = 214***		***Statistical analysis***	
						**P value**
	Frequency	Percent	Frequency	Percent		

					**Mann-Whitney U test**	
*Median*	3.0 μg/dL		16.0 μg/dL		−2.720	<0.0001*
*Inter - quartile range*	0–141		4–140			
	μg/dL		μg/dL		**Chi-square**	
*Normal*	135	63.1%	76	35.5%	32.539	<0.0001*
*Lead poisoning*	79	36.9%	138	64.5%		
***Exposure thresholds***						
*<10 μg/dl (unexposed / normal)*	135	63.1%	76	35.5%		
*10 – 40 μg/dl (acceptable)*	55	25.7%	105	49.1%	33.54	<0.0001*
*>40 μg/dl (dangerous)*	24	11.2%	33	15.4%		

^*^ Significant

**Table 4:** Symptoms Associated with Lead Poisoning Among Roadside and Organized Panel Beaters in Enugu Metropolis

***Variable***	***Blood lead <10 μg/dl***		***Blood lead* ≥*10 μg/dl***		***Statistical analysis***	
	Frequency	Percent	Frequency	Percent	**Chi - square**	**P value**

***Symptoms***						
***Roadside panel beaters***						
*Headache*	111	66.1%	57	33.9%	2.995	0.088
*Numbness*	3	25.0%	9	75.0%		0.010*
*Abdominal Colic*	67	64.4%	37	35.6%	0.156	0.777
*Nausea*	19	76.0%	6	24.0%	2.028	0.189
*Tremor*	2	40.0%	3	60.0%		0.361**
*Blue line*	1	50.0%	1	50.0%		1.000**
*Weakness*	115	62.8%	68	37.2%	0.032	1.000
*Fatigue*	68	71.6%	27	28.4%	5.294	0.023*
*Disturbed sleep*	7	50.0%	7	50.0%	1.101	0.391
*Drowsiness*	7	53.8%	6	46.2%	0.507	0.557
*Seizures*	1	33.3%	2	66.7%		0.556
*Forgetfulness*	2	28.6%	5	71.4%		0.103**
*Bone pains*	106	62.7%	63	37.3%	0.045	0.864
*Erectile dysfunction*	1	50.0%	1	50.0%		1.000**
***Organized panel beaters***						
*Headache*	49	35.0%	91	65.0%	0.047	0.881
*Numbness*	0	0.0%	2	100.0%		0.540**
*Abdominal Colic*	28	41.8%	39	58.2%	1.678	0.219
*Nausea*	5	18.5%	22	81.5%	3.897	0.054
*Tremor*	0	0.0%	1	100.0%		1.000**
*Blue line*	0	0.0%	1	100.0%		1.000**
*Weakness*	65	40.1%	97	59.9%	6.185	0.019*
*Fatigue*	48	41.7%	67	58.3%	4.206	0.046*
*Disturbed sleep*	3	15.0%	17	85.0%		0.051**
*Drowsiness*	4	22.2%	14	77.8%		0.305**
*Seizures*	1	20.0%	4	80.0%		0.658**
*Forgetfulness*	3	17.6%	14	82.4%		0.122**
*Bone pains*	64	38.3%	103	61.7%	2.620	0.122
*Erectile dysfunction*	2	22.2%	7	77.8%		0.497**

^*^Significant

^**^Fischer’s exact test

## Discussion

Due to the effects of Pb poisoning at varying thresholds, there is no entirely safe BLL. As Pb levels increase, the severity of damage to target organs and consequent symptoms increase.^6^ The current study found that median BLLs were within the normal and acceptable levels for roadside and organized panel beaters, with a higher prevalence of Pb levels ≥ 10 μg/dL among organized sector workers compared to roadside sector workers. Organized sector workers operate in enclosed outlets or warehouse-like workshops for vehicle repair with ineffective local ventilation control systems compared to the open spaces along roadsides that offer greater dilution of atmospheric Pb. This is in agreement with studies in Lagos, Nigeria, Kenya, Ethiopia, Thailand and South Korea where high prevalences of Pb poisoning have been found.^10,30,31,32,33,34^ This indicates that chronic exposures to Pb continue to be of public health concern among occupational groups across different thresholds coupled with weak or absent occupational health practices in low- or medium-income countries. Occupational exposures through various occupational processes of auto repair, cutting, soldering, spray painting, etc. contribute to “*take home Pb*” where children are secondarily exposed to Pb at home from their occupationally-exposed parents or adults siblings through inhalation or contact of Pb particles on clothes.^35,36,37^

The symptomatic effects associated with Pb poisoning in the present study were fatigue and numbness of the limbs among the roadside panel beaters, while weakness and fatigue were noted among organized panel beaters. Fatigue was the most common symptom associated with Pb poisoning in both groups of panel beaters. These are nonspecific constitutional symptoms and could be early warnings of Pb poisoning in the nervous, hematological, cardiovascular, gastrointestinal and renal system. This illustrates the high burden of subclinical effects of Pb poisoning at lower dose exposures and recognition of these symptoms will help in preventing workers from developing further irreversible effects of overt clinical toxicities. A study in Jimma town, Ethiopia among automotive garage workers with mean BLL of 19.75 μg/dL found that 50–80% of workers complained of headache, depression, and memory impairment and 40% complained of loss of appetite and nausea.^31^ A study in Sudan among used lead acid battery (ULAB) factory workers found that 50% complained of insomnia, fatigue, weakness and drowsiness, 41% complained of abdominal colic and constipation and 2% complained of blue lines on the gums.^38^ These studies similarly report the presence of nonspecific symptoms among occupational workers following Pb exposure. The symptoms reported by respondents in the present study are in keeping with the current understanding that low dose chronic Pb exposures cause damage to body systems and there is no safe level of Pb.^6,39^ Recognition of these nonspecific symptoms can help in the prevention of further Pb poisoning among occupationally exposed workers.

### Study limitations

This study is a cross-sectional study, and the symptoms experienced were limited to the period of study. All the participants in the present study were male. In addition, the study is specific to one area and therefore the results are not generalizable.

## Conclusions

Nonspecific constitutional symptoms are the most common manifestations of occupational Pb poisoning at lower dose exposures. These symptoms occurred irrespective of BLLs content among roadside and organized panel beaters. Symptoms included weakness, fatigue, nausea and numbness of the limbs. Employers and workers should be made aware of the importance of these nonspecific symptoms in their occupational practices through health education. Routine biological and environmental monitoring is recommended to employers to keep Pb exposures to below 10 ug/dL. Other safety practices and engineering controls should be enforced in the workplace.

### Funding

This study was funded as part of employment.

## References

[1] Omotosho I.O (2019). Oxidative Stress Indices as Markers of Lead and Cadmuim Exposure Toxicities in Automobile Technicians in Ibadan, Nigeria. Oxid Med Cell Longev.

[2] International Labor Organization (ILO) | NIGERIA Factories Act, 1987. https://www.ilo.org/dyn/natlex/docs/WEBTEXT/47979/65089/E87.

[3] International Labor Organization (ILO) | NIGERIA Employee's Compensation Act 2010. https://www.ilo.org/wcmsp5/groups/public/---ed_protect/---protrav/---ilo_aids/documents/legaldocument/wcms_172642.pdf.

[4] Anthony P (2013). Unorganized sector: Role of an Enterpreneur and Challenges in self. Employment International Journal of Scientific and Research Publications (IJSRP).

[5] Ukaejiofo EO, Thomas N, Ike SO (2009). Haematological assessment of occupational exposure to lead handlers in Enugu urban, Enugu State, Nigeria. Niger J Clin Pract.

[6] Flora G, Gupta D, Tiwari A (2012). Toxicity of lead : A review with recent updates. Interdiscip Toxicol.

[7] Landrigan P.J (1989). Editorial: Toxicity of lead at low dose. Brit J Ind Med.

[8] Needleman H (2004). Lead Poisoning. Annu Rev Med.

[9] Centre for Disease Control (CDC) Health Hazards Evaluation: Issues Realted to Occupational Exposure to Lead 1994 – 1999. DHHS (NIOSH) Publication 2001 – 113.

[10] Saliu A, Adebayo O, Kofoworola O, Babatunde O, Ismail A (2015). Comparative Assessment of Blood Lead Levels of Automobile Technicians in Organised and Roadside Garages in Lagos , Nigeria. Journal of environmental and public health (JEPH).

[11] Gidlow DA (2004). Lead toxicity. Occup Med – C.

[12] Tadashi S (2000). Biomarkers of Lead Exposure. Review Article Ind Health Industrial Health.

[13] World Health Organization (WHO) Lead Poisoning and Health 2019. https://www.who.int/news-room/fact-sheets/detail/lead-poisoning-and-health.

[14] Vorvolakos T, Arseniou S, Samakouri M (2016). There is no Safe Threshold of Lead Exposure. A literature review. Psychiatriki.

[15] Staudinger K.C, Roth V.S (1998). Occupational lead poisoning – American family physician. https://www.aafp.org/afp/1998/0215/p719.html.

[16] Center for Disease Control (CDC) | Advisory Committee on Childhood Lead Poisoning Prevention (2012). Low Level Lead Exposure Harms Children: A Renew Call for Primary Prevention. https://www.cdc.gov/nceh/lead/docs/final_document_030712.pdf.

[17] Kosnett M.J, Watson RT, Fuller M, Pokras M, Hunt WG (2009). Health effects of Low Dose Exposure in Adults and Children and Preventable Risk posed by the Consumption of Game Meat Harvested with Lead Ammunition. Ingestion of Lead from Spent Ammunition Implications for Wildlife and Humans The Peregrine Fund Boise Idaho USA.

[18] Tong S, von Schirnding YE, Prapamontol T (2000). Environmental lead exposure: a public health problem of global dimensions. B World Health Organ.

[19] Prüss-üstün A, Fewtrell L, Landrigan PJ, Ayuso-mateos JL (2004). Lead exposure: Comp Quantification of Health Risks: Global and Regional Burden of Disease Attributable to Selected Major Risk Factors. Publication of World Health Organization.

[20] Institute for Health Metrics and Evaluation (IHME) (2017). GBD compare. http://www.healthdata.org/data-visualization/gbd-compare.

[21] Institute for Health Metrics and Evaluation (IHME) GBD Compare | IHME Viz Hub. https://vizhub.healthdata.org/gbd-compare/.

[22] Levin R (2016). The Attributable Annual Health Cost of U.S. Occupational Lead Poisoning. Int J Occup Environ Health.

[23] Aniebue P, Aguwa E, Obi E (2010). Universal precautions: awareness and practice of patent medicines vendors in Enugu metropolis, South East Nigeria. Nigerian Medical Journal (NMJ) Medknow Publications (India).

[24] National Population Commission of Nigeria Nigerian Population Census 2016 [Internet]. http://www.population.gov.ng/.

[25] Enugu State South Eastern Region Economic Development company. https//Southeast.ng/seredec/the-region/enugustate/.

[26] Ofili AN, Usiholo EA, Oronsaye MO (2009). Psychological morbidity, job satisfaction and intentions to quit among teachers in private secondary schools in Edo-State, Nigeria. Ann Afr Med.

[27] Fewtrell L, Kautmann R, Pruss-ustun A Assessing the environmental burden of disease at national and local levels. http://www.who.int/quantifying_ehimpacts/publications/en/leadebd2.pdf.

[28] Massachusetts Division of Occupational Safety Medical Evaluation Questionnaire for Occupational Lead Exposure. https://lead.org.au/fs/fst66.html.

[29] Wani A.B, Ara A, Usman J.A (2015). Lead Toxicity: A Review. Interdiscip Toxicol.

[30] Ashraph JJ, Kinyua R, Mugambi F, Kalebi A (2003). Health effects of lead exposure among Jua Kali (informal sector) workers in Mombasa Kenya. International Journal of Medicine and Medical Science (IJMMS).

[31] Adela Y, Ambelu A, Tessema DA (2012). Occupational lead exposure among automotive garage workers - a case study for Jimma town, Ethiopia. J Occup Med Toxicol.

[32] Chamnong T, Alan T.G, Mark G.R (2007). Exposure to Lead of Boatyard workers in Southern Thailand. J Occup Health.

[33] Lormphongs S, Morioka I, Miyai N, Yamamoto H (2004). Occupational Health Education and Collaboration for Reducing the Risk of Lead Poisoning of Workers in a Battery Manufacturing Plant in Thailand. Ind Health Industrial Health.

[34] Kim Y, Lee H, Lee CR, Park DU, Yang JS, Park IJ (2002). Evaluation of lead exposure in workers at secondary lead smelters in south Korea: with focus on activity of erythrocyte pyrimidine 5 - nuclotidase (P5N). Sci Total Environ.

[35] Center for Disease Control (CDC) | Mortality and Morbidity weekly Report (MMWR) Take – Home Lead Exposure Among Children with Relatives Employed at a Battery Recycling Facility – Puerto Rico, 2011. https://www.cdc.gov/mmwr/preview/mmwrhtml/mm6147a4.htm.

[36] Occupational Safety and Health Administration (OSHA) | Quick card If You Work Around Lead, Don't Take it Home. https://www.osha.gov/Publications/OSHA3680.pdf.

[37] Rinsky J.L, Higgins S, Angelon – Gaetz K, Hogan D, Lauffer P, Davies M (2018). Occupational and Take – home Lead Exposure Among Lead Oxide Manufacturing Employees, North Carolina, 2016. Public Health Rep.

[38] El Karim A.A, Hamed A.S, Yusuf A.A, Elhaimi Y.A, Osman Y (1986). Effects of exposure to Lead among Lead acid battery factory workers in Sudan. Arch Environ Health.

[39] Njoroge JK, Njagi ENM, Orinda GO, Sekadde – Kigondu CB, Kayima JK (2008). Environmental and occupational exposure to lead. East Afr Med J.

