# Supplementary material for: Symptoms Associated with Low Threshold Lead Poisoning Among Roadside and Organized Panel Beaters in Enugu Metropolis, Nigeria
Source: J Health Pollut. 2021 Feb 25;11(29):210303. doi: 10.5696/2156-9614-11.29.210303 (PMC8009647; doi:10.5696/2156-9614-11.29.210303)
Supplement: Supplementary file 1 [file Kassy_Supplemental_Material.docx]

**Supplemental Material**

**RESEARCH QUESTIONNAIRE**

**CONFIDENTIAL**

**TITLE:** **Symptoms Associated with Low Threshold Lead Poisoning Among Roadside and Organized Panel Beaters in Enugu Metropolis, Nigeria.**

Dear Sir/Madam, I am a Public Health physician from the Department of Community Medicine, UNTH Enugu undertaking a study on the above-named title. Kindly answer the following questions. All information will be treated with utmost confidentiality. Thank you.

**SECTION A- IDENTIFICATION**

1. Questionnaire ID………………………………………………..
2. i. Organized sector/company No……………………………….

ii. Roadside sector/branch No………………………………….

1. Name of interviewer……………………………………………
2. Date of interview………………………………………………

**SECTION B- SOCIO-DEMOGRAPHIC INFORMATION**

1. Age ----------- years.
2. Sex: (a) Male [ ] (b) Female [ ]
3. Ethnicity: (a) Igbo [ ] (b) Other [ ]
4. Religion: (a) Christianity [ ] (b) Muslim [ ] (c) Traditional religion [ ]
5. Education: (a) None [ ] (b) Primary [ ] (c) Secondary [ ] (d) Tertiary [ ]
6. Marital status: (a) Single [ ] (b) Married [ ] (c) Separated [ ] (d) Divorced [ ]
7. How much do you earn in a month? -------------------------------------------------

**SECTION C – EFFECTS OF EXPOSURE TO LEAD**

Question 12 are on effects of exposure to lead. You are to tick the correct symptoms.

Which of the following illness are often felt during work.

Headache (a) Yes [ ] (b) No [ ]

Numbness of limbs (a) Yes [ ] (b) No [ ]

Abdominal colic pain (a) Yes [ ] (b) No [ ]

Nausea (a) Yes [ ] (b) No [ ]

Tremor (a) Yes [ ] (b) No [ ]

Lead or blue line on gum (a) Yes [ ] (b) No [ ]

Weakness (a) Yes [ ] (b) No [ ]

Fatigue (a) Yes [ ] (b) No [ ]

Disturbed sleep (a) Yes [ ] (b) No [ ]

Drowsiness (a) Yes [ ] (b) No [ ]

Seizures (a) Yes [ ] (b) No [ ]

Forgetfulness (a) Yes [ ] (b) No [ ]

Bone | joint pains (a) Yes [ ] (b) No [ ]

Erectile dysfunction | infertility (a) Yes [ ] (b) No [ ]

**SECTION D – LABORATORY LINE LISTING TABLE**

| Sample no | Date | Blood lead |
| --- | --- | --- |
